# NPNT is Expressed by Osteoblasts and Mediates Angiogenesis via the Activation of Extracellular Signal-regulated Kinase

**DOI:** 10.1038/srep36210

**Published:** 2016-10-26

**Authors:** Vincent Kuek, Zhifan Yang, Shek Man Chim, Sipin Zhu, Huazi Xu, Siu To Chow, Jennifer Tickner, Vicki Rosen, Wendy Erber, Xiucheng Li, Qin An, Yu Qian, Jiake Xu

**Affiliations:** 1 School of Pathology and Laboratory Medicine, University of Western Australia, Nedlands WA 6009, Australia; 2 Department of Orthopaedics, Shaoxing People’s Hospital, Shaoxing, Zhejiang 312000, China; 3 Department of Orthopaedics, The Second Affiliated Hospital, Wenzhou Medical University, Wenzhou, Zhejiang, 325035, China; 4 Developmental Biology, Harvard School of Dental Medicine, Boston, MA 02115, USA; 5 Shanghai Key Laboratory of Orthopaedic Implants, Department of Orthopaedic Surgery, Shanghai Ninth People’s Hospital, Shanghai Jiao Tong University School of Medicine, Shanghai, China

## Abstract

Angiogenesis plays an important role in bone development and remodeling and is mediated by a plethora of potential angiogenic factors. However, data regarding specific angiogenic factors that are secreted within the bone microenvironment to regulate osteoporosis is lacking. Here, we report that Nephronectin (NPNT), a member of the epidermal growth factor (EGF) repeat superfamily proteins and a homologue of EGFL6, is expressed in osteoblasts. Intriguingly, the gene expression of NPNT is reduced in the bone of C57BL/6J ovariectomised mice and in osteoporosis patients. In addition, the protein levels of NPNT and CD31 are also found to be reduced in the tibias of OVX mice. Exogenous addition of mouse recombinant NPNT on endothelial cells stimulates migration and tube-like structure formation *in vitro*. Furthermore, NPNT promotes angiogenesis in an *ex vivo* fetal mouse metatarsal angiogenesis assay. We show that NPNT stimulates the phosphorylation of extracellular signal-regulated kinase 1/2 (ERK1/2) and p38 mitogen-activated kinase (MAPK) in endothelial cells. Inhibition of ERK1/2 impaired NPNT-induced endothelial cell migration, tube-like structure formation and angiogenesis. Taken together, these results demonstrate that NPNT is a paracrine angiogenic factor and may play a role in pathological osteoporosis. This may lead to new targets for treatment of bone diseases and injuries.

Angiogenesis is intimately coupled with osteogenesis to mediate bone development, remodelling and repair. An interruption of this coupling process could lead to osteoporosis, a condition commonly caused by aging and post-menopausal oestrogen deficiency^1^. For instance, aging mice showed a reduction in CD31^high^ endothelial cells and is associated with a decline in osteoprogenitors and bone volume^2^. In addition, ovariectomised (OVX) mice showed a reduction in bone volume, which is accompanied by a reduction in blood vessels^3^. Although the interrelationship between angiogenesis and osteogenesis is critically important, the regulatory factors which mediate this complex process remain poorly understood.

Osteoblasts, osteoclasts and vascular endothelial cells are known to be the main contributors to the remodelling process within a vascularised structure called the bone remodelling compartment (BRC). These cells establish a crosstalk system to mediate bone cell activities and the recruitment, proliferation and differentiation of cells from mesenchymal and haematopoietic lineages^4^,^5^. Endothelial cells can regulate bone cells in a paracrine manner via the secretion of macrophage colony-stimulating factor (M-CSF), receptor activator of nuclear factor kappa-B Ligand (RANKL) and various other chemokines^6^,^7^,^8^. In contrast, osteoblasts produce angiogenic factors such as VEGF, BMP7, EGFL6 and TGF-α to mediate angiogenesis in the bone microenvironment^9^. Furthermore, osteoclasts and preosteoclasts have also been recently implicated in bone formation and angiogenesis by secreting MMP-9 and PDGF-BB, respectively^3^,^10^. Importantly, angiogenic factors such as VEGF play a crucial role in fracture healing and distraction osteogenesis^11^,^12^.

Nephronectin (NPNT) is a 70–90 KDa extracellular matrix protein originally identified in the embryonic kidney^13^. Previous studies report that NPNT is required for kidney and heart development^14^,^15^. Furthermore, NPNT was found to promote osteoblast differentiation via EGF-like repeats in MC3T3-E1 osteoblastic cells, and is regulated by TGF-β^16^,^17^. Interestingly, it shares a similar homology with EGFL6, an EGF-like protein which is involved in mediating the proliferation of human adipose tissue-derived stromal vascular cells and angiogenesis^18^,^19^,^20^. Many EGF-like protein family members such as EGF, HB-EGF and EGFL7 are known to be involved in promoting endothelial cell migration and angiogenesis^9^,^21^. Although NPNT contains EGF-like domains, its potential role in mediating angiogenesis in bone and osteoporosis remains to be elucidated. In this study, we examined the expression of NPNT in the bone local environment using *in vitro* and *in vivo* approaches. Furthermore, we characterized the role of NPNT on endothelial cell activities, angiogenesis and the signalling mechanisms involved using *in vitro* functional assays.

## Materials and Methods

### Cell culture

Osteoclasts were formed by treating primary C57BL/6J mouse bone marrow macrophages (BMM) with recombinant RANKL as previously described^21^. Osteoblasts were formed by culturing primary calvariae cells of neonatal C57BL/6J mice in osteogenic medium according to published protocols^21^,^22^. SVEC (a simian virus 40-transformed mouse microvascular endothelial cell line) and COS-7 (a monkey kidney fibroblast cell line) were cultured as previously described^21^.

### Real time reverse transcription (RT)-qPCR on osteoblasts

Total cellular RNA isolation and RT-PCR were performed as previously described^23^. qPCR amplification was carried out using SYBR green (Qiagen, Australia) and iCycler (BioRad) using the cycling parameters: 94 °C, 1 minute; 60 °C, 30 seconds; 72 °C, 45 seconds for 38 cycles with primers designed against the following mouse sequences: NPNT (forward: 5′-TGGGGACAGTGCCAACCTTTCT-3′; reverse, 5′-TGTGCTTACAGGGCCGAGGCT-3′), OCN (forward: 5′-GCGCTCTGTCTCTCTGACCT-3′; reverse: 5′-ACCTTATTGCCCTCCTGCTT-3′), Alkaline phosphatase (ALP) (forward: 5′-AACCCAGACACAAGCATTCC-3′; reverse: 5′-GCCTTTGAGGTTTTTGGTCA-3′), Col1A1 (forward: 5′-CTGGCGGTTCAGGTCCAAT-3′; reverse: 5′-TCCAAACCACTGAAGCCTCG-3′), 18S rRNA (forward: 5′-ACCATAAACGATGCCGACT-3′; reverse: 5′-TGTCAATCCTGTCCGTGTC-3′). RT-qPCR results were analysed by ViiA™ 7 Software.

### Western blotting

Western blotting was performed as previously described^21^. Immunoreactivity detection and quantification was performed as previously reported^18^,^21^. Additional detailed protocols and antibodies’ information are provided in the Supplementary Methods.

### Transfection and detection of NPNT in COS-7 cells

Transfection was performed according to protocols described previously^18^,^21^. Briefly, COS-7 cells were transfected with NPNT expression vector pcDNA3.1-NPNT-c-myc/His or empty pcDNA3.1 vector (control) using lipofectamine 1000 (Invitrogen, Australia). After 6 hours, cells were washed twice and incubated with Opti-MEM reduced serum medium (Gibco). Supernatants were harvested at 24 and 48 hours post-transfection and the presence of NPNT was examined by Western blotting using anti-c-myc antibody.

### Mouse ovariectomised model

Female C57BL/6J mice, 5 weeks old, were purchased from Shanghai Super–B&K laboratory animal Corp. Ltd (Shanghai, China). All the animal procedures were approved by the ethics committee (2015/081) of the Shaoxing People’s Hospital (Shaoxing, China). Animal experiments were performed in accordance with the approved guidelines. Animals were subjected to the following conditions: normal diet, 20–25 °C room temperature, 50–60% relative humidity, and 12 hours light/dark cycle. After acclimatizing for 2 weeks, mice were divided into sham group or OVX group randomly (6 per group). Mice in the OVX group received ovariectomies bilaterally, and only sham operations were performed in sham group. Animals in both groups were housed for another 3 months post-operation, followed by euthanasia. The forelimbs and femurs were harvested for microCT imaging and RT-qPCR (Supplementary Methods).

### Acquirement of patient samples

Ethical approval of all the procedures which involved human subjects was granted by the ethics committee (2015/082) of the Shaoxing People’s Hospital (Shaoxing, China). Informed consent was obtained from all the subjects involved, and experimental procedures were carried out in accordance with the approved guidelines. Caput femoris samples were collected from patients who received hip replacement following femoral neck fracture or hip osteoarthritis, and were stored at −80 °C until analyzed. These samples were divided into osteoporosis group (femoral neck fracture) and control group (osteoarthritis) (n = 20) for microCT imaging and RT-qPCR (Supplementary Methods). The detailed information of patients who participated in this study was listed in Supplementary Table 1.

### Immunohistochemistry (IHC)

The bone sections of mouse tibias were deparaffinised and rehydrated, and incubated with 1% hydrogen peroxidase in methanol for 30 minutes to block endogenous peroxidase activity. Non-specific binding sites were blocked with goat serum for 60 minutes at room temperature before incubation with primary antibody against NPNT (1:600, abcam) or CD31 (1:800, abcam) overnight at 4 °C. For detection, sections were incubated with HRP-conjugated secondary antibody for 60 minutes, followed by the addition of liquid DAB substrate. Sections were counterstained with haematoxylin and were dehydrated and mounted with fast drying mounting media (United Biosciences, Australia). Light microscope and image analysis software (Image plus 6.0) were used and five random regions of each section were taken for statistical analysis of IHC.

### *In vitro* angiogenesis assays

SVEC scratch-wound healing assay, SVEC tube-like structure formation assay and mouse fetal metatarsal angiogenesis assay were performed as previously described^21^. Recombinant mouse NPNT (R&D Systems), human bFGF (PeproTech, Inc.), human VEGF (PeproTech, Inc.) and ERK1/2 inhibitor U0126 (Promega) were purchased for the assays. Ethics approval was obtained from the UWA animal ethics committee (RA/3/100/1331) to carry out the metatarsal angiogenesis assay. Experiments were performed in accordance with the approved guidelines.

Additional detailed protocols are provided in the Supplementary Methods.

### Statistical analysis and data presentation

All data were presented as means + SD. For single comparisons, the difference between the two means was evaluated by unpaired, two-tailed Student’s t test. For comparison between multiple means, one-way ANOVA was conducted. A level of P < 0.05 was considered as statistically significant. Statistical analysis was performed using SPSS software version 19.0. Sample sizes in each animal and patient group were presented as n/group. All *in vitro* data shown represent one of at least three independent experiments.

## Results

### Gene expression profiles of NPNT during osteoblast and osteoclast differentiation

Previous study has reported that NPNT is expressed by MC3T3-E1 osteoblastic cell line and during the development of long bone and calvaria^16^, but its role in angiogenesis and osteoporosis is unknown. In the present study, we performed microarray analysis and found that NPNT is upregulated during primary osteoblast differentiation (day 7), along with the expression of osteoblast marker genes alkaline phosphatase (ALP), osteocalcin (OCN), bone sialoprotein (BSP) and high temperature requirement protease A (HtrA1), a common osteoblast/osteoclast marker^23^ (Supplementary Fig. 1A). However, NPNT expression was not altered during RANKL-induced osteoclast differentiation (day 5), whereas osteoclast-associated marker genes calcitonin receptor (CTR), cathepsin K (CtsK), dendritic cell-specific transmembrane protein (DC-STAMP) and HtrA1 were upregulated. Consistent with this observation, further analysis of RANKL-induced osteoclast gene expression by RT-PCR was unable to detect NPNT expression (Supplementary Fig. 1B). Multiple sequence alignment using UniProt (www.uniprot.org) revealed that NPNT’s domains are well conserved between mouse and human (Supplementary Fig. 1C). These include a signal peptide sequence at the N-terminus, EGF-like domain sequence, a RGD motif and MAM domain at the C-terminus. Further tree guide analysis also revealed that mouse NPNT is more closely related to human and orangutan NPNT compared to rat NPNT (Supplementary Fig. 1D).

**Figure 1:**
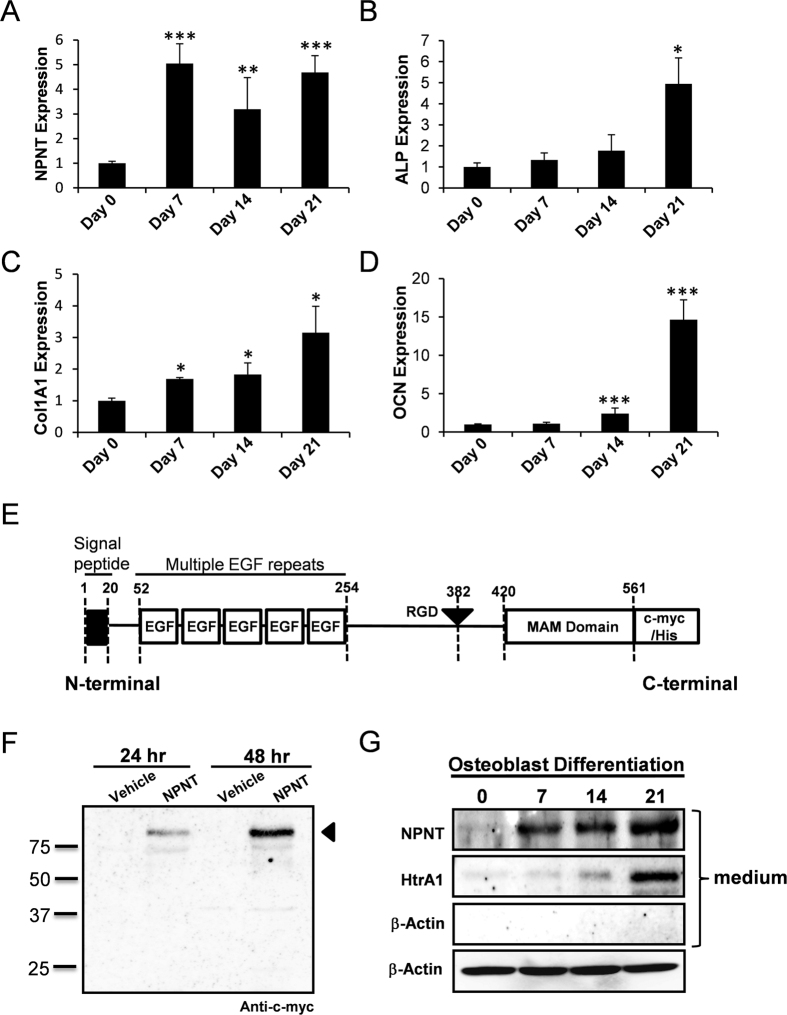
Expression patterns of NPNT in osteoblasts. (**A**) RT-qPCR was performed during osteoblast differentiation using primers specific for (**A**) NPNT, (**B**) ALP, (**C**) Col1A1, and (**D**) OCN. Gene expression was normalized to 18S and compared to the expression at day 0. (**E**) NPNT expression construct (pcDNA3.1-NPNT-c-myc/His), which encodes a mouse full length NPNT, was generated. (**F**) Detection of NPNT protein in the conditioned medium of COS-7 cells transfected with NPNT expression construct using anti-c-myc antibody. (**G**) NPNT was detected in osteoblast supernatants using anti-NPNT antibody. HtrA1 was used as positive marker for osteoblast differentiation and β-Actin was used as loading control. Western blot images are presented as cropped format. Full length blots are presented in Supplementary Fig. 4. *P < 0.05, **P < 0.01, ***P < 0.001.

To further investigate the pattern of NPNT gene expression in osteoblasts, we performed RT-qPCR on mRNAs extracted from primary calvarial osteoblasts during differentiation. We showed that NPNT mRNA levels are significantly higher on 7, 14 and 21 days compared to pre-culture with osteogenic differentiation medium on day 0 (Fig. 1A). The differentiation of osteoblasts was confirmed by the upregulation of established osteogenic markers ALP, OCN and alpha-1 type 1 collagen (Col1A1) (Fig. 1B–D).

### NPNT is a secreted factor by osteoblasts

Next, we examined osteoblast cell culture for NPNT expression via Western blot analysis. We found that the NPNT protein was present in the supernatant (Fig. 1G) and lysates (Supplementary Fig. 2B), indicating that NPNT is secreted by osteoblasts. Furthermore, an expression construct of mouse full length NPNT (pcDNA3.1-NPNT-c-myc/His) was generated and transiently transfected in COS-7 cells to express NPNT recombinant protein (Fig. 1E). NPNT was detected in the conditioned medium of COS-7 cells 24 and 48 hours post-transfection using anti-c-myc antibody (Fig. 1F), and also in the lysates (Supplementary Fig. 2A). This attested that NPNT is a secreted protein, consistent with other reports that NPNT is an extracellular matrix protein^13^,^20^.

### NPNT expression was down-regulated in OVX mice and osteoporotic patients

**Figure 2:**
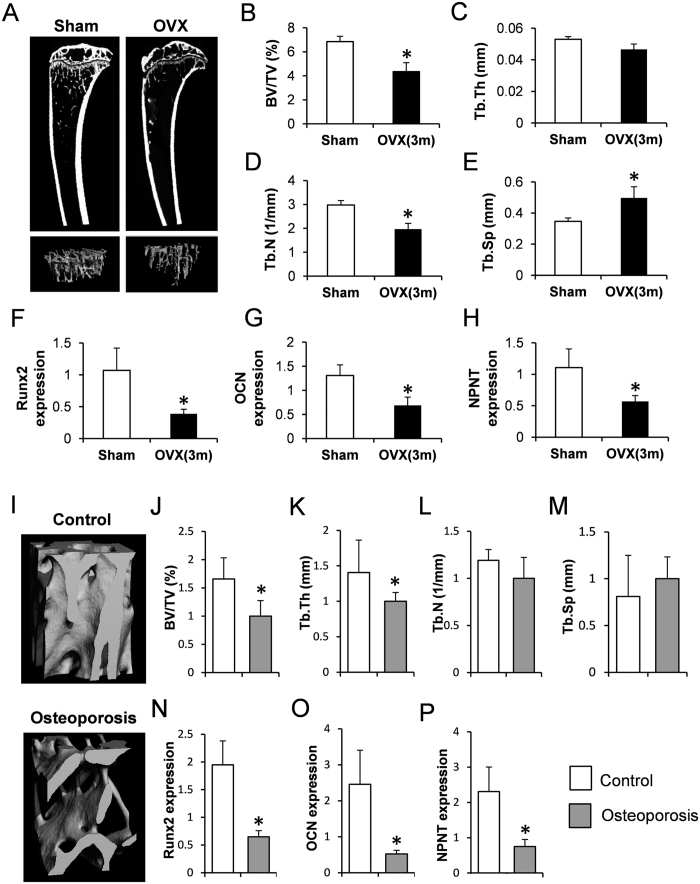
NPNT gene expression was down-regulated in OVX mice and in osteoporotic patients. (**A**) Tibias from sham group (left) and OVX group (right) were visualized by μCT scanning, and the ROI focused on the trabecular region below the growth plate for quantitative analysis of (**B**) Tb.BV/TV, (**C**) Tb.Th, (**D**) Tb.N and (**E**) Tb.Sp. Real-time PCR quantitative analysis of (**F**) Runx2, (**G**) OCN and (**H**) NPNT gene expression in mouse forelimbs. n = 6/group. (**I**) μCT images of trabecular bone in the region between head and neck of femur from patients with osteoarthritis (top) and osteoporosis (bottom), and quantitative analysis of (**J**) Tb.BV/TV, (**K**) Tb.Th, (**L**) Tb.N and (**M**) Tb.Sp. Real-time PCR quantitative analysis of (**N**) Runx2, (**O**) OCN and (**P**) NPNT gene expression in human femora. n = 20/group. The gene expressions were normalized to β-Actin and 18S. *P < 0.05.

Next, we studied NPNT gene expression using *in vivo* disease models. Firstly, we generated OVX mouse models to mimic the pathological condition post-menopausal osteoporosis in humans (Fig. 2A). In the OVX group at 3 months post-operation, Tb.BV/TV and Tb.N at the proximal tibia were markedly lower than in sham (control) littermates, with Tb.Sp significantly higher in the OVX group (Fig. 2B,D,E). In contrast, no significant difference in Tb.Th was detected between the groups (Fig. 2C). Subsequent RT-qPCR revealed a distinct reduction of NPNT expression in the OVX group (Fig. 2H), along with reductions in the expression of osteogenic markers Runx2 and OCN (Fig. 2F,G).

Next, we examined the NPNT gene expression in the trabecular bone samples obtained from clinical patients (Fig. 2I). Quantitative analysis revealed that Tb.BV/TV and Tb.Th were significantly lower in the osteoporosis group compared to the osteoarthritis (control) group (Fig. 2J,K), while no prominent difference was observed in Tb.N and Tb.Sp between the two groups (Fig. 2L,M). Moreover, RT-qPCR showed that the expressions of NPNT, along with Runx2 and OCN, were significantly lower in the osteoporotic patients in accord with the animal experiment results (Fig. 2N–P).

**Figure 3:**
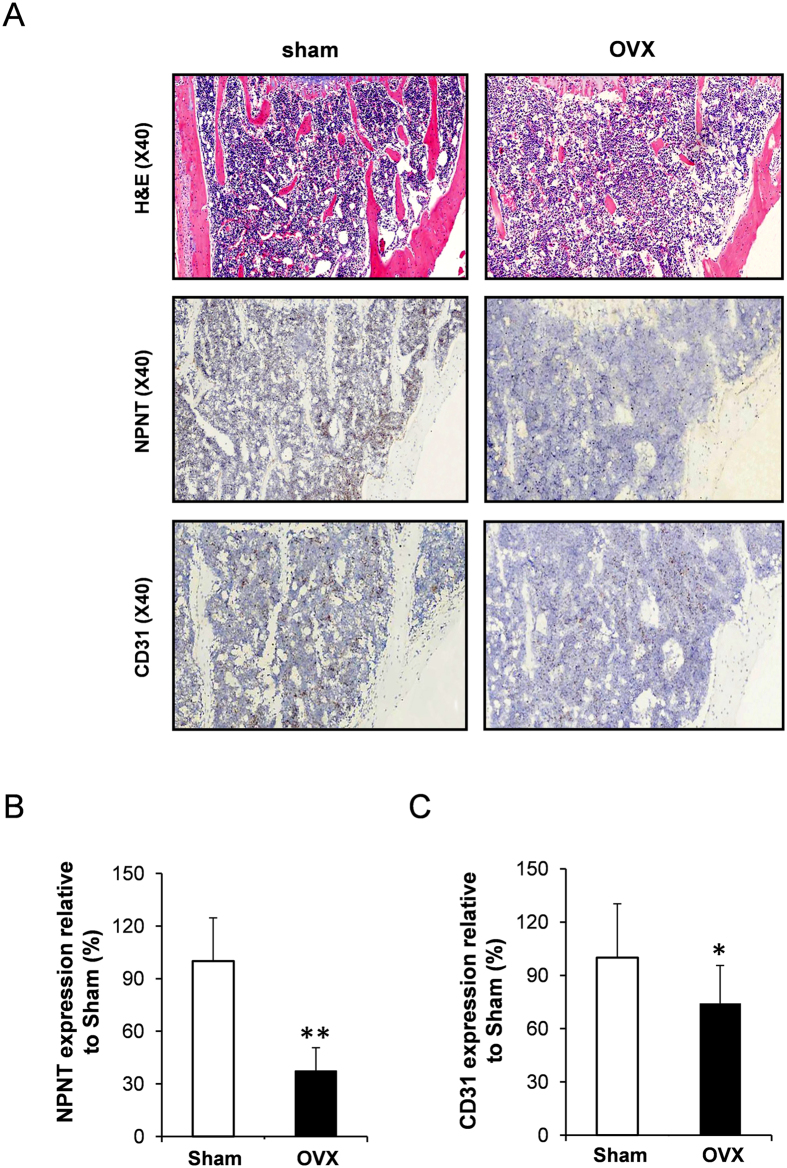
NPNT and CD31 protein expressions were reduced in the tibias of OVX mice. (**A**) Representative microscopic images of H&E and immunostaining using NPNT and CD31-specific antibody on tibia sections of sham and OVX mice. Quantitative analysis of (**B**) NPNT and (**C**) CD31 protein expressions in the tibias of OVX mice. *P < 0.05, **P < 0.01.

In addition, we performed immunostaining to examine the protein expression of NPNT and CD31 (an endothelial marker) in the tibias of both sham and OVX mice (Fig. 3A). We found that the protein expression of NPNT was significantly reduced in the tibias of OVX mice, which was consistent with the reduction of NPNT gene expression as described previously (Fig. 3B). Interestingly, we also found that the CD31 expression was significantly lower in the tibias of OVX mice compared to sham mice (Fig. 3C).

### Induction of cell migration and tube formation by SVEC endothelial cells and mouse fetal metatarsal angiogenesis by NPNT

**Figure 4:**
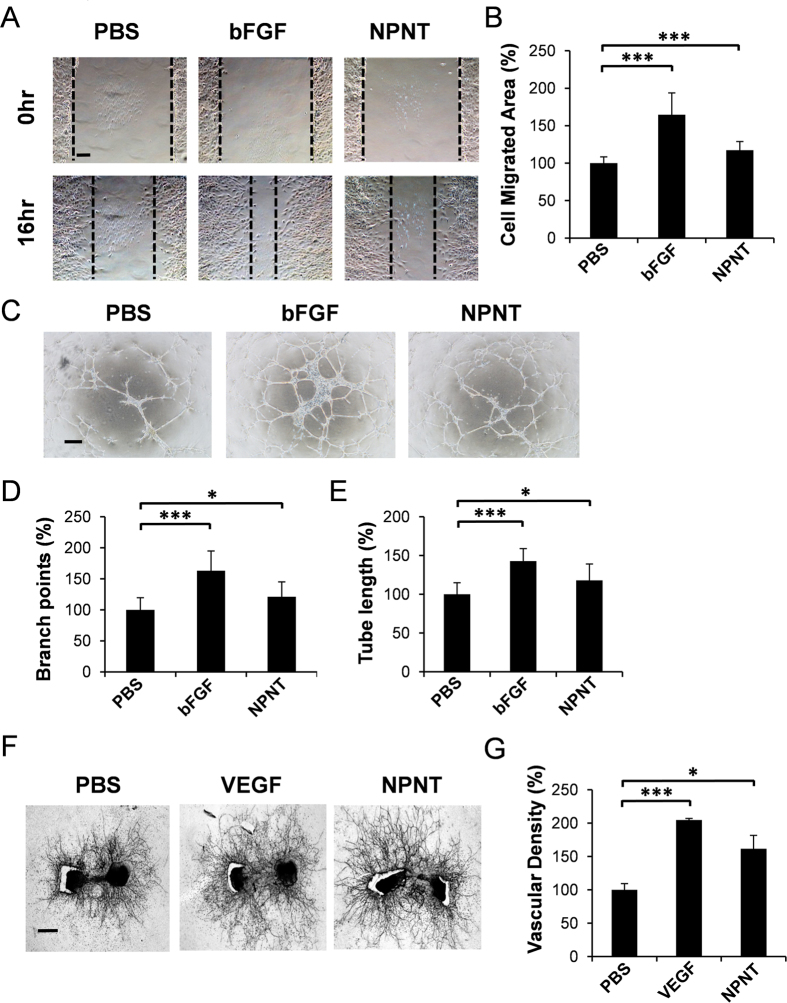
NPNT promotes endothelial cell migration, tube-like structure formation and angiogenesis. (**A**) Representative microscopic views of scratch wound healing assays performed using SVEC cells treated with recombinant mouse NPNT (500 ng/ml) from 0 to 16 hours. Scale bar, 100 μm. (**B**) Quantitative analysis of cell migration area. (**C**) Representative images showing tube-like structure formation by SVEC cells following treatment with recombinant mouse NPNT (500 ng/ml) for 24 hours. Scale bar, 100 μm. (**D**,**E**) Quantitative analysis of branch points and tube lengths. PBS and bFGF were used as a negative and positive control respectively. (**F**) Representative images showing that recombinant mouse NPNT (200 ng/ml) induced vessel outgrowth from metatarsals dissected from E17.5 embryos. Scale bar, 250 μm. (**G**) Quantitative analysis of vessel sprouting. PBS and VEGF (50 ng/ml) were used as a negative and positive control respectively. *P < 0.05, **P < 0.01, ***P < 0.001.

Previously, it has been reported that NPNT has sequence homology with EGFL6^20^. In view of this similarity, we sought to investigate the role of NPNT on endothelial cell activities and angiogenesis. Firstly, we examined the effects of NPNT on SVEC migration by scratch wound healing assay. As shown in Fig. 4A,B, exogenous addition of recombinant NPNT protein significantly enhanced endothelial cell migration by ~15% compared to PBS control. bFGF was used as a positive control.

The formation of a three-dimensional tube-like structure is an important step in angiogenesis. Here, we found that recombinant NPNT protein stimulated the formation of tube-like structure by SVEC cells, with the branch points and tube lengths significantly enhanced compared to PBS control (Fig. 4C–E). To validate the role of NPNT on angiogenesis, we further performed an *ex vivo* fetal mouse metatarsal angiogenesis assay. As shown in Fig. 4F,G, mouse recombinant NPNT significantly promoted the formation of web-like structures in cultured metatarsals.

### NPNT activates ERK1/2 and p-38 signalling pathways

**Figure 5:**
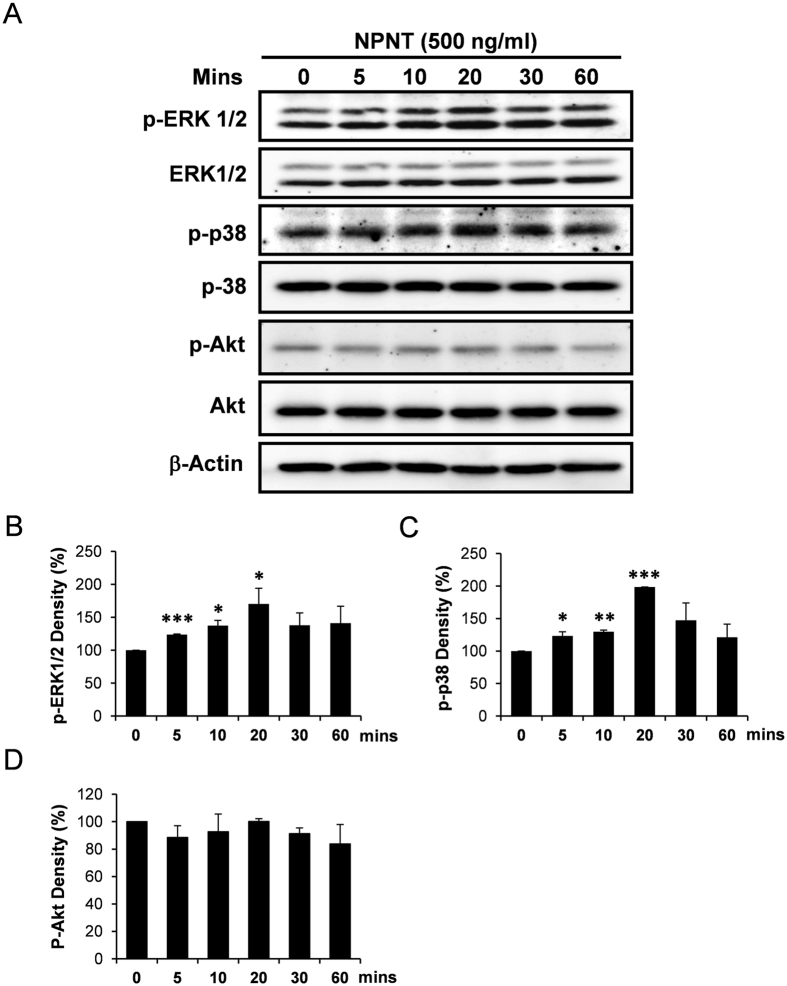
NPNT induced phosphorylation of ERK1/2 and p-38. (**A**) Western blot images showing the treatment of SVEC cells by mouse recombinant NPNT resulted in the phosphorylation of ERK1/2 and p-38, but not Akt. β-Actin was used as a loading control. (**B**–**D**) Quantification of signal intensities of p-ERK1/2, p-p38 and p-Akt by ImageJ. Induction ratios at each timepoint were compared to 0 minute, with p-ERK1/2 normalized to ERK1/2, p-p38 normalized to p-38 and p-Akt normalized to Akt. Western blot images are presented as cropped format. Full length blots are presented in Supplementary Fig. 4. *P < 0.05, **P < 0.01, ***P < 0.001.

To address the signalling mechanism of NPNT-induced endothelial cell activities, SVEC cells were stimulated with NPNT recombinant protein for 0, 5, 10, 20, 30 and 60 minutes, followed by Western blotting to detect the phosphorylation of key signalling molecules. As shown in Fig. 5A, NPNT induced the phosphorylation of ERK1/2 and p-38, whereas Akt remains relatively constant. The NPNT-induced phosphorylation of ERK1/2 and p-38 appears to be significantly upregulated compared to pre-stimulation (0 minute), with the levels peaking at 20 minutes (Fig. 5B,C). No significant change in the phosphorylation level of Akt was detected (Fig. 5D).

### U0126 impairs NPNT-mediated endothelial cell migration, tube-structure formation and metatarsal angiogenesis

**Figure 6:**
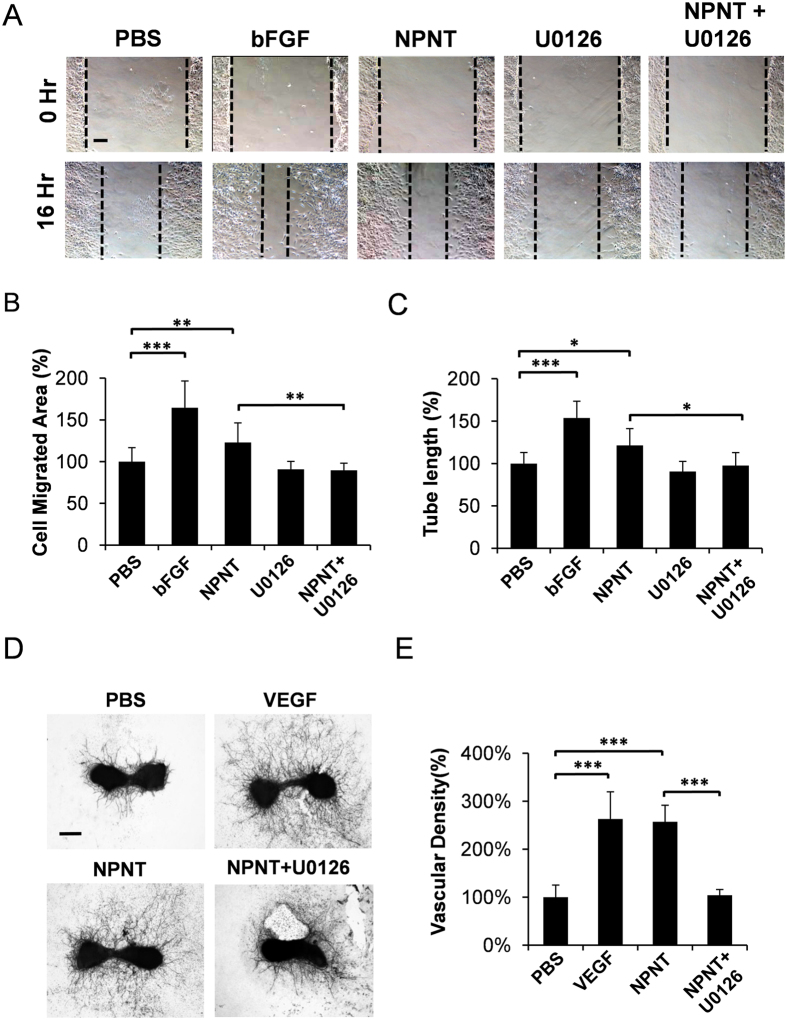
NPNT-induced endothelial cell activities and angiogenesis were inhibited by U0126. (**A**) Representative microscopic images of scratch wound healing assays showing NPNT-induced endothelial cell migration was blocked in the presence of U0126 (5 μM). Scale bar, 100 μm. Quantitative analyses showing that (**B**) NPNT-induced endothelial cell migration and (**C**) tube-like structure formation were significantly inhibited by U0126. PBS and bFGF were used as a negative and positive control, respectively. (**D**) Representative images showing that NPNT-induced angiogenesis was inhibited in the presence of U0126 (5 μM). Scale bar, 250 μm. (**E**) Quantitative analysis of vessel sprouting. *P < 0.05, **P < 0.01, ***P < 0.001.

Next, we investigated the effect of U0126 (MEK1/2 inhibitor) on NPNT-mediated endothelial cell functions as previously described^18^,^21^. As shown in Fig. 6A–C, NPNT-induced endothelial cell migration and tube-like structure formation was significantly inhibited in the presence of U0126. We also found that NPNT-induced blood vessel formation was significantly reduced in the presence of U0126 (Fig. 6D,E). Treatment with U0126 alone did not have significant effect on angiogenesis as compared to PBS control (data not shown), consistent with previous study^21^. The inhibition of NPNT-induced ERK1/2 phosphorylation by U0126 in the endothelial cells was confirmed via Western blot analysis (Supplementary Fig. 3).

## Discussion

Osteoporosis is often linked to reduced bone quality and increased risk of fracture. Importantly, an association between changes in the vascular architecture and loss of bone mass has been proposed in aged mice^2^, OVX mice^3^ and human primary osteoporosis^24^, suggesting that angiogenesis-osteogenesis coupling may be critical to maintain bone homeostasis. Therefore, identifying novel angiogenic factors in the bone microenvironment could expand our knowledge on the communication between bone cells and endothelial cells, and the roles that they might play in pathological bone diseases. In this study, we have shown that NPNT is expressed and secreted during the differentiation of osteoblasts. Furthermore, we found that the gene expression of NPNT is reduced in femur samples from OVX mice and in osteoporotic femora of humans. Importantly, this observation correlates to a reduction in bone mass and the reduced expression of key osteogenic markers, thus implying that NPNT is at least partly regulated by osteoblasts in the bone microenvironment. We also observed that the reduction of NPNT expression in bone of OVX mice is correlated to a reduction in CD31 expression via immunostaining, thus implying that CD31-expressing cells, including endothelial cells, may be regulated by NPNT.

Previously, our group has demonstrated that EGFL6 and EGFL7 promote angiogenesis^18^,^21^. Here, we showed that NPNT can also exhibit angiogenic potential by regulating endothelial cell migration, tube-structure formation and blood vessel growth from mouse fetal metatarsals. Moreover, we found that NPNT could regulate endothelial cell activities and angiogenesis through the activation of ERK1/2 and p38 MAPK signalling pathways. In fact, many EGF-like family members can mediate angiogenesis through the activation of MAPK pathways^18^,^25^. Interestingly, NPNT did not affect the phosphorylation of Akt, which plays a crucial role in cell survival and suppression of apoptosis^26^,^27^,^28^. This result appears to be similar to our previous study which demonstrated that EGFL6 activates ERK1/2, but not Akt signalling pathways in endothelial cells^18^. As NPNT shares a very similar structural homology with EGFL6, it is possible that both molecules could regulate angiogenesis in a spatio-temporal manner through similar signalling pathways. NPNT, unlike EGFL6, is a direct target of Wnt/β-catenin signalling^29^. NPNT expression is upregulated by Wnt/β-catenin activation, directly or via inhibition of BMP signaling. Furthermore, deletion of NPNT results in upregulation of EGFL6, thus suggesting a compensatory regulation between the two proteins^29^. Interestingly, NPNT is expressed in SVEC endothelial cells, but not EGFL6 (data not shown).

EGF-like proteins contain domains which are highly homologous to EGF. This is a common feature among the EGF-like protein family members such as HB-EGF, betacellulin, and amphiregulin^9^. It has been demonstrated that many EGF-like family members are expressed in osteoblasts and osteoclasts^9^, and their roles in regulating angiogenesis have been implicated^25^,^30^,^31^,^32^,^33^. Therefore, it is possible that NPNT could regulate angiogenesis via the same mechanisms as other EGF-like members. In fact, NPNT contains an RGD motif, which is critical in binding integrins^13^,^34^,^35^. Indeed, NPNT has been reported to be a major ligand for α_8_β_1_ integrin, and has lower binding affinities to other integrins such as α_v_β_3_, α_v_β_5_, α_v_β_6_ and α_4_β_7_^13^. Although the data regarding the presence of α_8_β_1_ integrin on endothelial cells is currently lacking, many vascular-related integrin receptors have been reported on endothelial cells, including α_1_β_1_, α_2_β_1_^36^, α_v_β_5_^37^, α_5_β_1_^38^ and α_v_β_3_^39^. In addition, previous studies have demonstrated that EGFL7, a potent EGF-like angiogenic factor, mediates angiogenesis through the binding of α_v_β_3_ integrin and the activation of integrin-associated signalling cascades^21^,^40^. Therefore, future studies could explore the possibility that NPNT could regulate endothelial cell activities through integrin interaction.

Many bone diseases have been reported to be associated with disruptions and changes in vascularisation^9^. Furthermore, angiogenesis plays a crucial role during the healing process of bone fractures and growth plate injuries^41^,^42^. Therefore, identifying new angiogenic factors may facilitate the development of new therapeutic treatments for skeletal pathologies and injuries. More recently, our group has identified EGFL2, EGFL3, EGFL5, EGFL6, EGFL7, EGFL8 and EGFL9 to be differentially expressed in the bone microenvironment, of which EGFL6 and EGFL7 play an important role in regulating angiogenesis^18^,^21^. Our current study presents evidence, for the first time, that NPNT gene expression is affected in osteoporosis and could potentiate angiogenesis *in vitro*. Therefore, it is plausible to postulate that NPNT could mediate the coupling process between angiogenesis and osteogenesis in the bone microenvironment. Future studies will be required to address the *in vivo* role of NPNT in bone using genetically-modified mouse models.

In summary, we have shown that NPNT is expressed by osteoblasts and its expression is reduced in osteoporosis. Furthermore, we also found that NPNT has a direct effect on endothelial cell activities and regulates angiogenesis via p-38 and ERK pathways. This study demonstrates the role of NPNT as a potentially important molecule in the communication between osteoblasts and endothelial cells by a paracrine mode of action. These data may lead to the development of novel therapeutic treatments for bone diseases and fractures.

## Additional Information

**How to cite this article**: Kuek, V. *et al*. NPNT is Expressed by Osteoblasts and Mediates Angiogenesis via the Activation of Extracellular Signal-regulated Kinase. *Sci. Rep*. **6**, 36210; doi: 10.1038/srep36210 (2016).

**Publisher’s note**: Springer Nature remains neutral with regard to jurisdictional claims in published maps and institutional affiliations.

## Supplementary Material

Supplementary Information
