# Corrigendum: NPNT is Expressed by Osteoblasts and Mediates Angiogenesis via the Activation of Extracellular Signal-regulated Kinase

**DOI:** 10.1038/srep37482

**Published:** 2016-11-21

**Authors:** Vincent Kuek, Zhifan Yang, Shek Man Chim, Sipin Zhu, Huazi Xu, Siu To Chow, Jennifer Tickner, Vicki Rosen, Wendy Erber, Xiucheng Li, An Qin, Yu Qian, Jiake Xu

Scientific Reports 6: Article number: 36210; 10.1038/srep36210 published online: 10 26 2016; updated: 11 21 2016.

The original version of this Article contained an error in the spelling of the author An Qin, which was incorrectly given as Qin An.

This has now been corrected in the PDF and HTML versions of the Article.

The Author Contributions Statement,

Conception and design: V.K., S.M.C., Z.Y., Y.Q., Q.A. and J.X. Acquisition and analysis of data: V.K., S.M.C., Z.Y., S.T.C., Y.Q., X.L., Q.A. and J.X. Drafting manuscript: V.K., S.M.C., Z.Y. and J.X. Revising manuscript: V.K., S.M.C., Z.Y., S.Z., H.X., Y.Q., Q.A., J.T., W.E., V.R. and J.X.

now reads:

Conception and design: V.K., S.M.C., Z.Y., Y.Q., A.Q. and J.X. Acquisition and analysis of data: V.K., S.M.C., Z.Y., S.T.C., Y.Q., X.L., A.Q. and J.X. Drafting manuscript: V.K., S.M.C., Z.Y. and J.X. Revising manuscript: V.K., S.M.C., Z.Y., S.Z., H.X., Y.Q., A.Q., J.T., W.E., V.R. and J.X.

