# Supplementary material for: NPNT is Expressed by Osteoblasts and Mediates Angiogenesis via the Activation of Extracellular Signal-regulated Kinase
Source: Sci Rep. 2016 Oct 26;6:36210. doi: 10.1038/srep36210 (PMC5080588; doi:10.1038/srep36210)
Supplement: Supplementary Information [file srep36210-s1.pdf]

NPNT is Expressed by Osteoblasts and Mediates Angiogenesis via the Activation of Extracellular Signal-regulated Kinase

Vincent Kuek[1*], Zhifan Yang[2*], Shek Man Chim[1*], Sipin Zhu[1, 3], Huazi Xu[3], Siu To Chow[1], Jennifer Tickner[1], Vicki Rosen[5], Wendy Erber[1], Xiucheng Li[2], Qin An[4], Yu Qian[2#], Jiake Xu[1,3#].

1. School of Pathology and Laboratory Medicine, University of Western Australia, Nedlands WA 6009, Australia;
2. Department of Orthopaedics, Shaoxing People's Hospital, Shaoxing, Zhejiang 312000 China.
3. Department of Orthopaedics, The Second Affiliated Hospital, Wenzhou Medical University, Wenzhou, Zhejiang, 325035 China.
4. Shanghai Key Laboratory of Orthopaedic Implants, Department of Orthopaedic Surgery, Shanghai Ninth People’s Hospital, Shanghai Jiao Tong University School of Medicine, Shanghai, China.
5. Developmental Biology, Harvard School of Dental Medicine, Boston, MA 02115.

Corresponding Author: Prof. Jiake Xu, School of Pathology and Laboratory Medicine , The University of Western Australia M508, 35 Stirling Hwy, Crawley, WA, 6009, Australia, Tel: +61 8 9346 2739; Fax: +61 8 9346 2891; E-mail: jiake.xu@uwa.edu.au; or Dr. Qian Yu. E-mail: doctor120@hotmail.com .

*Authors make equal contribution to this work.

Attached: Supplementary informations

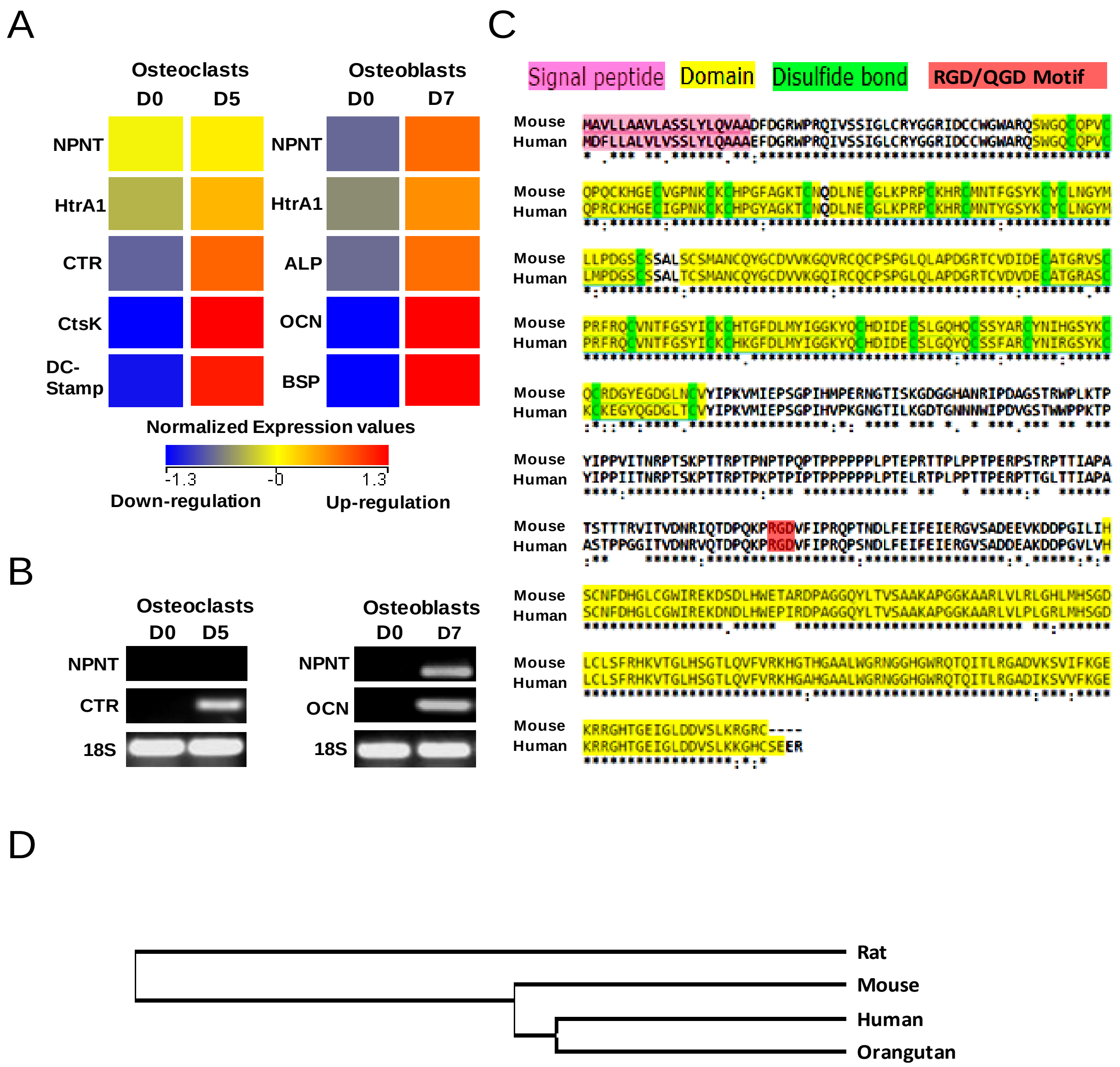

<u>Supplementary Figure 1.</u> NPNT gene expression during osteoclast and osteoblast differentiation and bioinformatics analysis among different species. (A) Total RNAs from BMM cells (Day 0), mature osteoclasts (Day 5), primary calvarial cells before (Day 0) and after (Day 7) differentiation were harvested for microarray analysis. Heatmap demonstrating the upregulation of NPNT in primary osteoblasts but not osteoclasts during differentiation, with osteoblast and osteoclast specific genes. (B) RT-PCR amplification confirming the specific expression of NPNT in osteoblasts. (C) Multiple alignment of full length mouse and human NPNT protein sequences. (D) Tree guide analysis of NPNT among different species.

A

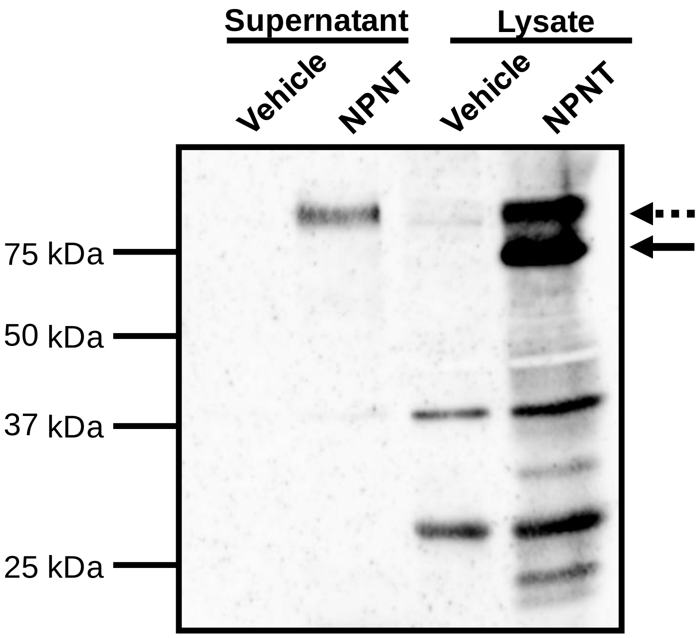

B

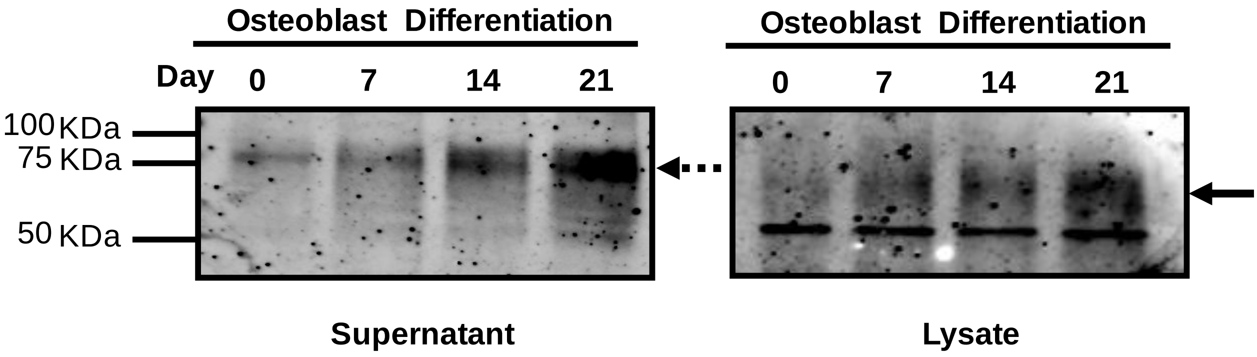

<u>**Supplementary Figure 2.**</u> **NPNT protein expression in osteoblasts and in COS-7 cells transfected with NPNT expression construct.** (A) NPNT protein expression was detected using anti-c-myc antibody in both the lysate and supernatant 72 hours post-transfection with NPNT expression construct, pcDNA3.1-NPNT-c-myc/His in COS-7 cells. The higher band (dotted arrow) indicates possible post-translational glycosylation of NPNT, compared to the lower band (solid arrow). (B) The expression of NPNT protein was also detected in the supernatant (dotted arrow) and lysate (solid arrow) of osteoblasts.

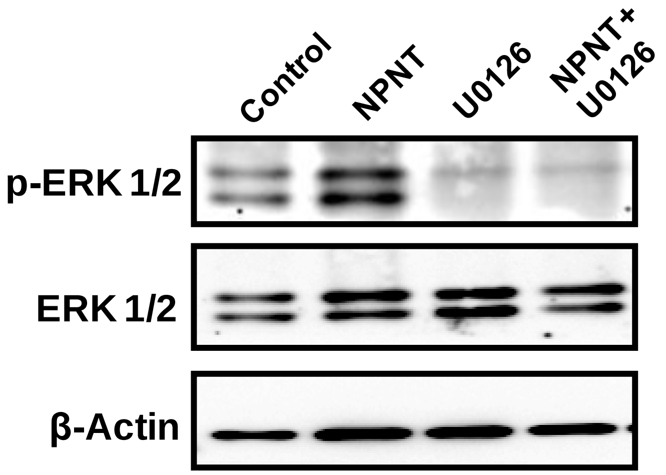

<u>Supplementary Figure 3.</u> **NPNT-induced ERK1/2 phosphorylation was inhibited by U0126, an ERK1/2 inhibitor.** Western blot images showing the inhibition of NPNT-induced ERK1/2 phosphorylation in the SVEC cells following treatment with U0126 (5µM). From left: Control (untreated), NPNT treatment, U0126 treatment and NPNT+U0126 treatment.

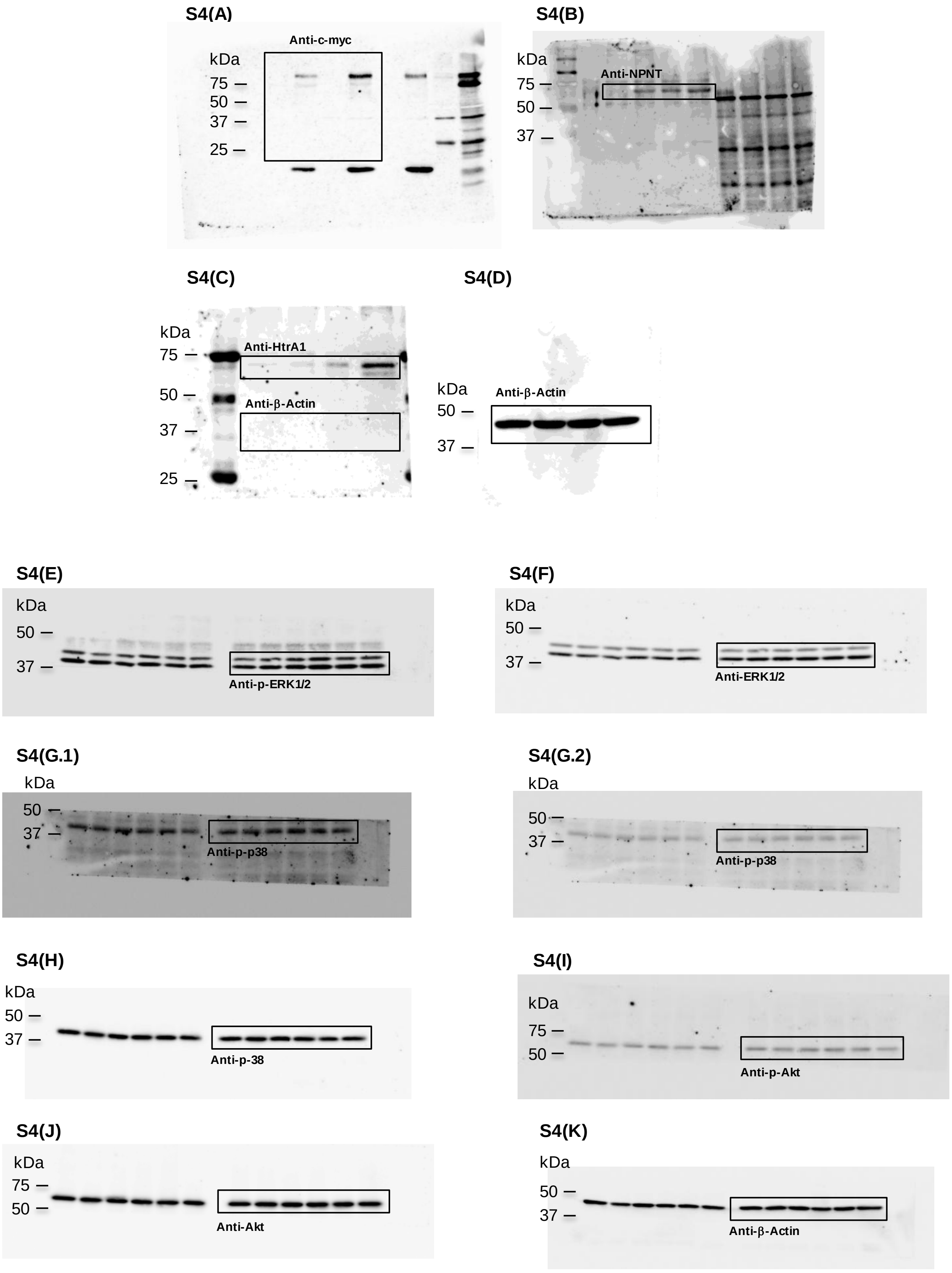
S4(A)
Anti-c-myc
kDa
75
50
37
25
S4(B)
kDa
Anti-NPNT
75
50
37
S4(C)
kDa
75
Anti-HtrA1
50
Anti-β-Actin
37
25
S4(D)
kDa
Anti-β-Actin
50
37
S4(E)
kDa
50
37
Anti-p-ERK1/2
S4(F)
kDa
50
37
Anti-ERK1/2
S4(G.1)
kDa
50
37
Anti-p-p38
S4(G.2)
kDa
50
37
Anti-p-p38
S4(H)
kDa
50
37
Anti-p-38
S4(I)
kDa
75
50
Anti-p-Akt
S4(J)
kDa
75
50
Anti-Akt
S4(K)
kDa
50
37
Anti-β-Actin

<u>Supplementary Figure 4.</u> Uncropped blots for the images shown in main Figures 1 and 5. Following membrane transfer, some membranes were cut into slices according to the molecular weight of the proteins of interest, and incubated with appropriate antibodies for the revelation. This is to ensure the economization of antibodies and reagents by simultaneously revealing several proteins on the same membrane. S4(A) Uncropped blot for image shown in Figure 1F. S4(B-D) Uncropped blots for images shown in Figure 1G. As β-Actin is a cytoplasmic protein, incubation with anti-β-Actin did not reveal any band in the osteoblast medium S4(C) at the approximate position of 42 kDA, but revealed distinct bands in the cellular lysates S4(D), as expected. S4(E-K) Uncropped blots for images shown in Figure 5A. Note that S4(G.1) and S4(G.2) are two different levels of exposure of the same membrane, with the higher exposure image S4(G.1) being shown in the main figures. All blot images shown here are uncropped and unprocessed. Molecular weight markers are inserted in the blot images, as previously reported[1,2].

	Control	Osteoporosis
Number of participants	20	20
Age, mean (range)	63.3 (36-81)	73.8 (56-84)
Gender, male/female	10/10	7/13
Diagnosis (N)	hip osteoarthritis (20)	femoral neck fracture (20)
Underlying disease (N)	hypertension (9), atrial fibrillation (1)	hypertension (11), atrial fibrillation (1)
	fatty liver (2), hepatitis B (2)	hepatitis B (2)
	SLE (1), AS (1)	atelectasis (1)
	diabetes (3), hyperlipidemia (1)	diabetes (4)
	cerebral infarction (2)	cerebral infarction (4), stroke (1)

SLE = Systemic Lupus Erythematosus, AS = Ankylosing Spondylitis

Supplementary Table 1. Background of patients who participated in this study.

Supplementary Methods

Osteoclast cell culture

Primary mouse bone marrow macrophages (BMM) were isolated from the marrow cavity of femora and tibia of C57BL/6J mice and filtered through a 100μm mesh as previously described[2]. The cells were cultured in α-modified Eagle's medium (α-MEM; Gibco, Australia), supplemented with 10% (v/v) fetal bovine serum (FBS; Gibco), 100U/mL Penicillin / 100μg/mL Streptomycin (Gibco), 1% Glutamax (Gibco) (complete α-MEM) and 10ng/ml M-CSF (R&D Systems, Australia). BMMs were maintained in 37°C and 5% CO2 incubator. To form mature osteoclasts, BMMs were cultured for 5 days in the presence of 50ng/ml recombinant RANKL protein expressed and purified as described previously[3].

Osteoblast cell culture

Primary mouse osteoblasts were prepared from the calvariae of neonatal C57BL/6J mice. Enzymatic digestions were performed according to published protocols[4]. Cells were seeded and cultured in Dulbecco's modified Eagle medium (DMEM; Gibco, Australia) supplemented with 10% FBS, 100U/mL penicillin / 100μg/mL Streptomycin (complete DMEM). Upon reaching confluence, cell medium was replaced with osteogenic differentiation medium, complete DMEM containing 50μg/ml ascorbic acid (Sigma-Aldrich), 10mM β-glycerophosphate (Sigma-Aldrich), and 10nM dexamethasone (Sigma-Aldrich), with fresh medium replacement every 2-3 days for 21 days as previously described[2].

SVEC and COS-7 cell culture

SVEC and COS-7 cell lines were cultured in complete DMEM (supplemented with supplemented with 10% FBS, 100U/mL penicillin / 100µg/mL Streptomycin).

RNA isolation and cDNA microarray

Total cellular RNA was extracted from osteoblast and osteoclast cultures using RNeasy Mini Kit (Qiagen, Australia) in accordance with the manufacturer's protocol. RNA concentration was determined by measuring the absorbance at 260nm with a Nano-drop 2000 (Thermo Scientific), and quality assessed using Agilent Bioanalyzer. Gene expression of NPNT was detected by cDNA microarray using mouse expression WG6 beadchip (Illumina, Inc.) according to the manufacturer's protocol.

Semi quantitative reverse transcription (RT)-PCR

Single-stranded cDNA was reverse transcribed from 2µg total RNA using reverse transcriptase with an oligo-DT primer. For semi quantitative RT-PCR, 1µl of each cDNA was amplified using cycling parameters 94°C, 40 seconds; 55°C, 40 seconds; 72°C, 40 seconds for 30 cycles with primers designed against the following mouse sequences: NPNT (forward: 5'-TGGGGACAGTGCCAACCTTTCT-3'; reverse, 5'-TGTGCTTACAGGGCCGAGGCT-3'), Calcitonin receptor (CTR) (forward: 5'-CGGACTTTGACACAGCAGAA-3'; reverse: 5'-CAGCAATCGACAAGGAGTGA-3'), Osteocalcin (OCN) (forward: 5'-GCGCTCTGTCTCTCGTGACCT-3'; reverse: 5'-ATAGATGCGTTTGTAGGCGG-3'), 18S rRNA (forward: 5'-ACCATAAACGATGCCGACT-3'; reverse: 5'-TGTCAATCCTGTCCGTGTC-3'). Samples were analysed by DNA agarose gel electrophoresis.

Western blotting and antibodies

Total cellular proteins were extracted from cultured cells using RIPA lysis buffer. Cell lysates were centrifuged at 16000g for 20 minutes at 4°C and post-nuclear supernatants were collected. Equivalent amounts of extracted proteins were diluted in SDS-sampling buffer with 5% β-mercaptoethanol and incubated at 95°C for 5 minutes. Samples were then resolved by SDS-PAGE and transferred onto nitrocellulose membranes via electroblotting. Membranes were then blocked with 5% skim milk for 1 hour and probed with primary antibodies overnight at 4°C. Primary antibodies used were mouse-specific goat polyclonal anti-NPNT, rabbit polyclonal anti-HtrA1, mouse monoclonal anti-p-p-38, mouse monoclonal anti-p-ERK (Santa Cruz Biotechnology, CA), rabbit polyclonal anti-ERK, (Promega, Australia), rabbit polyclonal anti-p-38, rabbit polyclonal anti-p-Akt, rabbit polyclonal anti-Akt (Cell signalling Technology, Australia), mouse monoclonal anti-β-actin (JLA20; DSHB University of Iowa) and mouse monoclonal anti-c-myc (Sigma-Aldrich). All antibodies were used at the concentrations recommended by the suppliers. Membranes were washed and incubated with HRP-conjugated secondary antibodies (Sigma-Aldrich) for 1 hour. Immunoreactivity detection was performed using the Western Lightning Ultra (PerkinElmer, Australia) and FujiFilm Las-4000 Gel Documentation System (FujiFilm, Australia). Intensities of signal were quantified by NIH ImageJ software.

Micro-CT imaging

Ex vivo high-resolution μCT (μCT100, Scanco Bruttisellen, Switzerland) was used to quantify trabecula from patients and tibias harvested from sham/OVX mice at 3 months post-operation. The

samples were fixed in 4% paraformaldehyde for 48 hours, followed by infusion of 70% ethanol. The scanning parameters were 70 kVp and 200 μA, with a resolution of 10.0 μm per pixel. Image reconstruction software (μCT V6.1), data analysis software (μCT Evaluation Program V6.5) and three-dimensional model visualization software (μCT Ray V4.0) were used for further analysis. In the animal experiments, the region of interest (ROI) was selected starting from 5% of tibial length proximal to growth plate and extended proximally for another 10% of tibial length. Metaphyseal trabecular bones were analyzed to determine the trabecular bone volume fraction (Tb.BV/TV), trabecular thickness (Tb.Th), trabecular number (Tb.N), and trabecular spacing (Tb.Sp). ROI in patients' samples focused on a small mass of trabecular bone dissected from the region between the head and neck of the femur, with the same parameters under analysis.

RT-qPCR of mouse OVX and human patient samples

Total RNAs were extracted from patients' Caput femoris or mouse forelimb samples using RNAiso plus reagent (Takara, Dalian, China) according to the manufacturer's protocol. RNAs were then reverse transcribed to cDNA using a PrimerScript RT reagent kit (Takara, Dalian, China). RT-qPCR was performed to measure the quantitative expression of mRNA levels using SYBR Premix Ex Taq^{TM} (Takara, Dalian, China). The fluorescence of double-stranded products was monitored and data were normalized to internal housekeeping genes, beta-actin and 18S ribosomal RNA (18s). The PCR conditions were as follows: 94°C, 5 minutes, followed by 35 cycles of: 94°C, 15 seconds; 60°C, 15 seconds; 72°C, 20 seconds. The primer sequences are listed below:

Mouse: Runx2 (forward 5'-CCAGGCAGGTGCTTCAGAACTG-3'; reverse 5'-ACATGCCGAGGGACATGCCTGA-3'), OCN (forward 5'-ATCTTTCTGCTCACTCTGCT-3';

reverse 5'- CTACCTTATTGCCCTCCTG -3'), NPNT (forward 5'-GAAGCCTCGGCCCTGTAAG-3'; reverse 5'-AGCATGTATCCGTTGAGACAGTA-3'), 18s (forward 5'-CTGGATACCGCAGCTAGGAA-3'); reverse 5'- GAATTTCACCTCTAGCGGCG -3'), beta-actin (forward 5'-GATCTGGCACCACACCTTCT-3'; reverse 5'-GGGGTGTTGAAGGTCTCAAA-3').

Human: Runx2 (forward 5'-AGTTCCCAAGCATTTCATC-3'; reverse 5'-GGCAGGTAGGTGTGGTAGT-3'), OCN (forward 5'-CACACTCCTCGCCCTATT-3'; reverse 5'-GGTCTCTTCACTACCTCGCT-3'), NPNT (forward 5'-GTAAGCACAGGTGCATGAACA-3'); reverse 5'-GAACCATCCGGCATGAGCATA-3'), 18s (forward 5'-ACTCAACACGGGAAACCT-3'; reverse 5'-CGCTCCACCAACTAAGAA-3'), beta-actin (forward 5'-GATCTGGCACCACACCTTCT-3'; reverse 5'-GGGGTGTTGAAGGTCTCAAA-3').

Scratch-wound healing assay

Scratch-wound healing assay was performed as previously described[2]. In brief, SVEC were cultured to confluence, followed by serum starvation with Opti-MEM medium overnight prior to wounding. Cells were incubated at 37°C for 16 hours with Opti-MEM medium containing recombinant mouse NPNT (R&D Systems) (500ng/ml) or human bFGF (PeproTech, Inc.) as a positive control. Time-lapse images were captured at 0 and 16-hour time points in the same position using a Nikon Eclipse TE2000-5 microscope. Images of wound area were quantified using Nikon NIS-Elements computer software. For scratch-wound healing assay with ERK1/2 inhibitor U0126 (Promega), SVEC monolayers were treated with inhibitors for 1 hour prior to wounding.

Tube formation assay

Tube-like structure formation assay was performed as previously described[2]. SVEC cells were seeded onto the layer of Geltrex™ (a reduced growth factor basement membrane matrix) (Invitrogen) and cultured for 24 hours with Opti-MEM medium containing recombinant mouse NPNT or human bFGF as a positive control. Five randomly selected fields of view were captured using a Nikon Eclipse TE2000-5 microscope. Quantification of branch points and tube length was performed by Nikon NIS-Elements computer software.

Mouse fetal metatarsal angiogenesis assay

Metatarsal angiogenesis assay was performed as previously described[2]. In brief, metatarsals were dissected from E17.5 C57BL/6J mouse embryos and cultured in 24-well plates in complete α-MEM for 72 hours. The explants were cultured with mouse recombinant NPNT (200ng/ml) in the presence or absence of U0126 for 14 days, followed by fixation and staining with anti-CD31 rabbit polyclonal antibody (Abcam, Australia). Human VEGF (PeproTech, Inc.) (50ng/ml) was used as a positive control. Images were captured by Nikon Eclipse TE2000-5 microscope and quantified by NIH ImageJ software.

Supplementary References

1 Chim, S. M. *et al.* EGFL6 promotes endothelial cell migration and angiogenesis through the activation of extracellular signal-regulated kinase. *J Biol Chem.* **286**, 22035-22046 (2011).

2 Chim, S. M. *et al.* EGFL7 is expressed in bone microenvironment and promotes angiogenesis via ERK, STAT3, and integrin signaling cascades. *J Cell Physiol.* **230**, 82-94 (2015).

3 Xu, J. *et al.* Cloning, sequencing, and functional characterization of the rat homologue of receptor activator of NF-kappaB ligand. *J Bone Miner Res.* **15**, 2178-2186 (2000).

4 Bakker, A. D. & Klein-Nulend, J. Osteoblast isolation from murine calvaria and long bones. *Methods Mol Biol.* **816**, 19-29 (2012).
